# Metabolic Reprogramming in HIV+ CD4^+^ T-Cells: Implications for Immune Dysfunction and Therapeutic Targets in *M. tuberculosis* Co-Infection

**DOI:** 10.3390/metabo15050285

**Published:** 2025-04-22

**Authors:** Suheena Ayrga, Gerrit Koorsen

**Affiliations:** Department of Biochemistry, University of Johannesburg, Auckland Park, Johannesburg 2006, South Africa

**Keywords:** metabolomics, CD4^+^ T cells, *Mycobacterium tuberculosis*, HIV, immunometabolism, metabolic pathways

## Abstract

**Background/Objectives:** HIV and *Mycobacterium tuberculosis* (*M.tb*) co-infection presents a major global health burden. The immune response to *M.tb* is largely orchestrated by cluster of differentiation 4-positive (CD4^+^) T cells, with CD8^+^ T cells playing an auxiliary role. This study aims to investigate the immunometabolic response of CD4^+^ and CD8^+^ T cells to *M.tb* antigens, analysed using metabolomics, to elucidate metabolic shifts that may influence immune function in an HIV+ environment. **Methods:** Whole blood samples from newly diagnosed, treatment-naïve HIV+ individuals were stimulated with *M.tb* antigens early secreted antigenic target 6 (ESAT-6) and culture filtrate protein 10 (CFP-10) using the QuantiFERON^®^ (QFT) Gold Plus assay. Following incubation, plasma samples were analysed through untargeted nuclear magnetic resonance (1^H^-NMR) spectroscopy. Metabolomic data were processed using MetaboAnalyst, with differential metabolites identified through multivariate statistical analyses. **Results:** Metabolic profiling of PBMCs revealed distinct differences in response to *M.tb* antigens between CD4^+^ and CD4^+^/CD8^+^ T-cell activation. CD4^+^ T cells exhibited enhanced glycolysis, with elevated levels of metabolites that are linked largely to the Warburg effect. Additionally, vitamin D levels were found to correlate with certain metabolites, suggesting a role in modulating immune responses. **Conclusions:** These findings suggest a complex interplay between immune cell metabolism and activation in HIV+ individuals. The study demonstrates that HIV and *M.tb* co-infection significantly influences the broader metabolic profile of peripheral blood mononuclear cells (PBMCs), highlighting the altered metabolic pathways that are critical in immune responses and disease progression. These findings contribute to the understanding of immunometabolism in co-infection and emphasise the need for further research into targeted metabolic interventions.

## 1. Introduction

*Mycobacterium tuberculosis (M.tb*) is a bacterial pathogen capable of causing active and latent tuberculosis (LTB) infection [1]. LTB is a clinical syndrome that occurs after a patient is exposed to *M.tb*, the infection has been established, and an immune response has been generated to control the pathogen and force it into latency [1]. In the latent stage, the *M.tb* is contained within a granuloma and will stay dormant without any clinical signs or symptoms [2]. A granuloma is an organised and compact immunological structure composed of immune cells, including macrophages, monocytes, dendritic cells, neutrophils, epithelioid cells, foamy macrophages, and multi-nucleated T cells [3]. CD4^+^ and CD8^+^ T cells play a central role in maintaining the granuloma [3,4].

The immune system is activated when T cells recognise the major histocompatibility complexes (MHC) on the surface of antigen-presenting cells (APC) of viruses and bacteria [5]. CD4^+^ T cells are imperative for immune protection [6] and provide helper signals that induce the adaptive immune response [7]. CD4^+^ T cells assist B cells in making antibodies that induce the macrophages to develop an enhanced microbial activity that recruits neutrophils, eosinophils, and basophils to sites of infection [6]. CD4^+^ T cells also enable APCs to activate naïve CD8^+^ T cells, leading to the formation of many cytotoxic T cells [7] and the cell-mediated immune response to foreign antigens [8].

Previous research indicates that CD4^+^ T cells are the target T cells for human immunodeficiency virus (HIV) infection [9]. In the context of LTB and granuloma maintenance, CD4^+^ T-cell loss increases the risk of progressing from LTB to active TB [10,11]. CD8^+^ T cells initiate an immune response against foreign pathogens by recognising infected cells through MHC-1-dependent enzymes [12]. HIV’s high mutation rate enables it to evade CD8^+^ T-cell recognition by downregulating surface MHC-1 expression through infected CD4^+^ T cells [12]. This suggests that CD4^+^ T cells are essential in *M.tb* granuloma maintenance. Conversely, an increase in CD8^+^ T cells has been associated with active TB [13].

Vitamin D plays a crucial role in modulating both innate and adaptive immune responses, particularly in the context of HIV and *M.tb* co-infection [14]. It enhances antimicrobial activity by promoting antimicrobial peptide production and regulating cytokines, both essential for granuloma maintenance [15,16]. Vitamin D deficiency, common in HIV+ individuals, is linked to increased susceptibility to infections, including tuberculosis [17,18,19]. Lower vitamin D levels in HIV/*M.tb* co-infected individuals impair antimicrobial defences [20]. The absence of IFN-γ production in vitamin D-deficient HIV+ individuals further increases *M.tb* susceptibility, underscoring its importance in immune defence [21]. The vitamin D receptor (VDR) is activated by the active form of vitamin D (1,25(OH)_2_D_3_) [22,23]. The VDR is primarily involved in regulating cellular metabolism, but it also influences immune-related activities, such as antimicrobial defence and the induction of T cells [24].

While antiretroviral therapy has greatly increased the life expectancy of individuals living with HIV, management of this chronic health condition is affected by infection with *M.tb*. TB remains one of the leading causes of death in individuals living with HIV [25]. Studies have shown that in an individual co-infected with HIV and LTB (HIV/LTB), the *M.tb* bacterial load is increased [26,27,28,29]. The pathology of HIV/LTB co-infection remains poorly understood. Metabolomics has interestingly revealed complex alterations in the host metabolic pathways as a result of infection with or treatment of HIV and *M.tb* [30]. Several studies report alterations in glucose, protein and lipid metabolism in response to HIV [31,32,33], while changes in protein metabolism are particularly noted in response to *M.tb* infection [34]. While metabolomics has been used to study HIV and *M.tb* separately, the combined impact of HIV and *M.tb* on the metabolic profiles of CD4^+^ and CD8^+^ T cells in co-infection remains largely unexplored. Given the interaction between metabolism and immune function [35], here we used metabolomics to study the role of CD4^+^ T cells only vs CD4^+^/CD8^+^ T cells in the immune response to *M.tb* in the presence of HIV. Prior research has established that HIV significantly depletes CD4^+^ T cells and weakens their ability to recognise and respond to *M.tb* [36]. This study further supports these findings by demonstrating metabolic impairments in CD4^+^ T cells, particularly increased glycolysis and lactic acid accumulation, which contribute to immune exhaustion and facilitate HIV persistence.

Results showed that the T-cell response to *M.tb* antigens, particularly in the context of the Warburg effect, exhibits significant differences between the CD4^+^ and CD4^+^/CD8^+^ T-cell response. Specifically, key metabolites, such as glucose, pyruvic acid, lactic acid, glutamic acid, glycine, and L-alanine, were identified as significantly altered in response to *M.tb* antigens. The findings suggest a complex relationship between immune dysregulation and metabolic pathways in HIV+ individuals, emphasising the critical roles of both CD4^+^ and CD8^+^ T cells in the immune response to *M.tb*.

## 2. Materials and Methods

### 2.1. Sample Collection

Participants for the study were newly diagnosed, treatment-naive, HIV-positive (HIV+) individuals who self-reported never being diagnosed with TB. The samples were collected and processed immediately after diagnosis, and the duration of HIV infection prior to diagnosis was unknown. By selecting treatment-naïve individuals, this study aimed to capture the direct effects of HIV and *M.tb* on T-cell metabolism without antiretroviral therapy-induced confounders. All participants were of black African ancestry and were between 18 and 59 years old at the time of collection. A volume of 4 mL of venous blood was collected in 2 lithium heparin tubes from 51 individuals.

### 2.2. Initiating an Immune Response

IFN-γ was selected for this study because of its pivotal role in T-cell activation and immune defence, particularly in response to HIV and *M.tb*. Since IFN-γ is directly involved in CD4^+^ and CD8^+^ T-cell activation in the context of HIV and TB, its measurement provided the most relevant insights into the immune response of the study population. To assess the cell-type-specific immune response to *M.tb* from CD4^+^ T cells and CD8^+^ T cells, respectively, the QuantiFERON^®^ (QFT) Gold Plus kit (Qiagen GmbH, Qiagen Strasse 1, Hilden, Germany) was used to conduct the Interferon Gamma Release Assay (IGRA) according to the manufacturer’s details. IGRA stimulates a cell-mediated immune response in PBMCs by exposing the cells to *M.tb*-specific peptide antigens absent from all Bacillus Calmette–Guérin (BCG) strains of mycobacteria. The QFT Plus kit employs two distinct antigen tubes. The TB1 tube contains peptides from the ESAT-6 and CFP-10 proteins that elicit T-cell-mediated immune responses from CD4^+^ T-helper cells. The TB2 tube contains additional peptides from the *M.tb.* antigens ESAT-6 and CFP-10 proteins that induce cell-mediated immune responses from CD8^+^ cytotoxic T cells. A total of 1 mL of blood was transferred into each QFT-Plus tube directly after collection, mixed, and incubated at 37 °C for 24 h to stimulate an immune response. Following incubation, the plasma was separated from the PBMCs by centrifugation at 3 000 x g for 15 min and used to quantify the metabolic signature of the CD4^+^ and CD8^+^ T-cell response, the interferon γ (INF-γ) levels and circulating 25(OH)D_3_ levels.

### 2.3. 1^H^ NMR Spectroscopy Method for CD4^+^ T-Cell Response

Samples from the CD4^+^ immune response were outsourced, and the following protocol was used. The NMR buffer solution consisted of 1.5 M potassium phosphate buffer (pH 7.4) in deuterium oxide and 5.805 mM of trimethylsilyl-2,2,3,3-tetradeuteropropionic acid (TSP; Sigma-Aldrich, St. Louis, MO, USA). A trace amount of sodium azide was included in the buffer to prevent bacterial growth in the sample. Samples were filtered using Amicon Ultra—2 mL centrifugal units with 10 kDa membrane filters (Merck; Ref UFC201024). Each centrifugal filter unit was pre-rinsed three times using ~2 mL dH20 and centrifuged at 4500× *g* for 10 min to remove trace amounts of glycerol and glycerine from membrane filters, which can interfere with NMR signals. A volume of 100 µL of serum was then filtered using the pre-washed filters, which were centrifuged at 4500× *g* for 20 min. Filtered samples were prepared in a 2 mm NMR tube (outside Ø 2.0 mm, inside Ø 1.6 mm, length 100 mm) using the eVol^®^ NMR digital syringe and a 180 mm long bevel-tipped needle. The programmed pipetting sequence of the eVol^®^ NMR digital syringe was as follows: (1) aspirate 6 µL NMR buffer solution (1.5 M potassium phosphate solution in deuterium oxide with internal standard TSP (trimethylsilyl-2,2,3,3-tetradeuteropropionic acid (0.5805 mM); pH 7.4); (2) aspirate 54 µL of the filtered sample—maintaining the 10:90% ratio of D2O: H_2_O as per standard protocol; (3) purge 60 µL (this dispenses a prepared sample into a 2 mm NMR tube); (4) aspirate 60 µL; (5) purge 60 µL (mix sample once inside the 2 mm NMR tube to ensure homogeneity); followed by a wash sequence; (6) aspirate 100 µL distilled water; (7) purge 100 µL (waste); (8) aspirate 100 µL distilled water; (9) purge 100 µL (waste); (10) aspirate 100 µL distilled water; (11) purge 100 µL (waste). The Bruker MATCH system, consisting of an adapter with a gripper to hold the 2 mm NMR tube inserted into a 10 mm spinner, was used. Each NMR MATCH assembly was loaded onto a SampleXpress autosampler for NMR analysis [37]. Samples were measured at 500 MHz on a Bruker Avance III HD NMR spectrometer equipped with a triple-resonance inverse (TXI) 1^H^{15N, 13C} probe head and x, y, z gradient coils. 1^H^ spectra were acquired as 128 transients in 32 K data points with a spectral width of 6000 Hz and acquisition time of 2.72 s. Receiver gain was set to 90.5. The sample temperature was maintained at 300 K, and the H_2_O resonance was saturated by single-frequency irradiation during a relaxation delay of 4 s, with a 90° excitation pulse of 8 μs. Shimming of the sample was performed automatically on the deuterium signal. Fourier transformation and phase and baseline correction were done automatically. The spectra were individually uploaded onto www.bayesil.ca with the following parameters: plasma (filtered), TSP (5850 µM) as the chemical shift reference, 500 MHz, and standard speed for metabolite identification. The concentration (µM) of each metabolite was then exported into an Excel spreadsheet.

### 2.4. 1^H^ NMR Spectroscopy Method for CD4^+^/CD8^+^ T-Cell Response

Before NMR spectroscopy, 750 µL plasma samples from the ESAT-6 and CFP-10 antigen-containing IGRA tubes were heat-inactivated for 30 min at 56 °C to inactivate viable viral particles. The samples were subjected to ultrafiltration through 0.5 μL filters (Merck, Modderfontein, South Africa) to remove any macromolecules that could disrupt the quantification of metabolites. Before filtration, the ultra-0.5 μL filters were rinsed seven times with dH_2_O and centrifuged at 1028× *g* at 4 °C for 20 min to remove any residual glycerol bound to the filter membrane. The sample filtrate (350 μL) was diluted in 250 μL phosphate buffer (PBS, pH 7.4, Invitrogen, Carlsbad, CA, USA) composed of 20% (*v*/*v*) deuterium oxide (Sigma Aldrich, St Louis, MA, USA), 4% (*w*/*v*) sodium azide (Sigma Aldrich, St Louis, MA, USA) and 0.4% (*v*/*v*) trimethylsilyl propionate (TSP; Sigma Aldrich, USA). A volume of 600 μL of the solution was transferred into a 5 mm NMR tube (Bruker, Bremen, Germany). The 1D NOESY spectrum was acquired at 25 °C with an acquisition time of 1.64 s, a mixing time of 0.05 s, a relaxation delay of 2 s, and 128 scans performed with a receiver gain of 203 dB and a spectrum width of 10,000 Hz (20 ppm was recorded). The spectra were phase-corrected and baseline-corrected to the TSP reference signal at 0.00 ppm. The spectra were individually uploaded onto www.bayesil.ca with the following parameters: plasma (filtered), TSP as the chemical shift reference (9800 µM), 500 MHz, and standard speed for metabolite identification. The concentration (µM) of each metabolite was then exported into an Excel spreadsheet.

### 2.5. Metabolite Annotation and Data Analysis

The raw spectrum was uploaded to the Bayesil platform (https://www.bayesil.ca, accessed on 14 December 2024), where automated spectral processing steps—including zero-filling, Fourier transformation, phasing, baseline correction, smoothing, chemical shift referencing, and deconvolution—were applied. The resulting data matrix, comprising metabolite concentrations from CD4^+^ and CD4^+^/CD8^+^ T-cell responses, was analysed using MetaboAnalyst (v6.0; https://www.metaboanalyst.ca/MetaboAnalyst, accessed on 18 December 2024) for chemometric and functional analyses. Prior to modelling, data were normalised by sum and pareto-scaled, with four strong outliers (samples 3, 14, 44, and 53) removed after inspection. Chemometric models, including principal component analysis (PCA) and partial least-squares discriminant analysis (PLS-DA), were employed to identify metabolites responsible for group separation and assess the metabolic profiles of CD4^+^ and CD8^+^ T-cell responses. PCA highlighted trends, reduced data dimensionality, and identified potential outliers, while PLS-DA provided supervised group separation and predictive analyses. Model quality was assessed using cumulative R2 and Q2 values, where values >0.5 indicated validity. Following PLS-DA, variable importance in projection (VIP) scores identified key metabolites, with a threshold VIP ≥ 1.0 indicating a significant contribution to the model. Additionally, independent sample *t*-tests and false discovery rates (FDR < 0.050) were used to validate metabolite distinctions between groups. Statistical tests were conducted to assess whether age, sex, smoking history, and other demographic factors influenced the observed differences, and based on the results, they were ruled out. Through careful participant selection, exclusion of other co-morbidities, and a focused analysis of treatment-naïve individuals, the study minimised the influence of underlying conditions. Pathway analysis of identified metabolites was performed using MetaboAnalyst’s functional analysis module, which integrates Kyoto Encyclopedia of Genes and Genomes (KEGG) metabolic pathways for pathway enrichment and topology analyses. Enrichment analysis employed the hypergeometric test, while topology analysis used relative betweenness node centrality. While multiple metabolic pathways were identified, those with the highest number of metabolites present in the dataset were considered the most prominent. This suggests that while alternative pathways contribute to immune cell metabolism, the selected pathway remains the most prominent feature of T-cell activation in response to *M.tb* antigens. This framework enabled mapping of the metabolomic landscape and provided insights into metabolite clusters and key cellular signaling and metabolic networks. The findings support biological interpretation of observed metabolic changes, offering a detailed understanding of immune response mechanisms.

### 2.6. Validation Analysis

To ensure that the observed differences in T-cell responses were biological rather than instrument-related, a validation study was conducted on the same instrument. The results confirmed that these observed differences were indeed biological and not due to instrumental variability, as detailed in Supplementary Section S1.

### 2.7. INF-γ Quantification

INF-γ levels released from PBMCs in response to CD4^+^ and CD8^+^ T-cell activation were quantified using the QFT Gold Plus ELISA kit (Qiagen GmbH, Qiagen Strasse 1, Hilden, Germany) according to the manufacturer’s specifications. Optical density (OD) was measured on a microplate reader using the 450 nm filter with a 620–650 nm reference filter and used to calculate INF-γ levels utilizing the QFT Plus Analysis Software (v2.71.2 Build 06, Qiagen, Hilden, Germany). LTB status was assigned based on the INF-γ levels (LTB- < 0.35 IU/mL; LTB+ > 0.35 IU/mL).

### 2.8. 25(OH)D_3_ Quantification

Circulating 25(OH)D_3_ levels were quantified using the vitamin D ELISA (Elabscience, Houston, TX, USA) according to the manufacturer’s specifications. OD was measured at 450 nm on the Multiskan FC Microplate Reader (Thermo Fischer Scientific Inc., Waltham, MA, USA). GraphPad Prism (version 9.4.1) was used to plot a four-parameter logistic curve on a log–log graph, with standard concentration on the *x*-axis and OD values on the *y*-axis. For the analysis, the concentration values were converted to log values for the x-axis. The 25(OH)D_3_ concentration data distribution was assessed using the one-sample Kolmogorov–Smirnov test. None of the samples gave a normal Gaussian distribution. As such, the non-parametric Mann–Whitney U test was performed to investigate whether 25(OH)D_3_ concentrations differed significantly between the participants. Statistical analysis was performed using IBM^®^ SPSS^®^ Statistics (v 28.0.1.1) for Windows (SPSS Inc., Chicago, IL, USA). A *p*-value of ≤0.050 was considered significant.

### 2.9. VDR Methylation Analysis

PBMCs were extracted via density gradient centrifugation, and DNA was extracted using the Qiagen DNeasy^®^ kit (Qiagen GmbH, Qiagen Strasse 1, Hilden, Germany) and concentrated using the DNA Concentrator Kit (Abcam, Cambridge, UK). Bisulfite conversion of DNA was performed using the EZ DNA Methylation-Lightning™ kit (Zymo Research, Irvine, CA, USA). Methylation-sensitive high-resolution melting (HRM) analysis targeted the VDR CGI 1060 region, with primers designed to amplify a 167 bp product. A standard curve was developed using methylated and unmethylated DNA standards to quantify methylation levels. qPCR was performed, and melt curves were analysed using Precision Melt Analysis™ software (v 4.0.52.0602, Bio-Rad, Johannesburg, South Africa). Statistical analysis involved non-parametric tests, including Spearman’s correlation, to assess the relationship between methylation levels and plasma 25(OH)D_3_ concentrations, with significant differences determined by a *p*-value < 0.05.

## 3. Results

### 3.1. Distinct Metabolic Profiles Distinguish Between a CD4^+^ T-Cell Only, and a CD4^+^ Plus CD8^+^ T-Cell Response to M.tb

**Figure 1:**
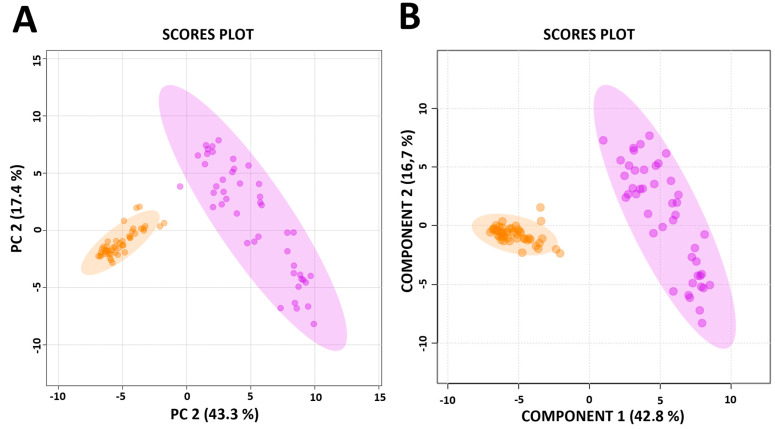
(**A**) Principal component analysis of CD4^+^ and CD4^+^/CD8^+^ T cell responses to *Mycobacterium tuberculosis* (*M.tb*) antigens early secreted antigenic target 6 (ESAT-6) and culture filtrate protein 10 (CFP-10). The plot depicts the multivariate modelling of nuclear magnetic resonance (1^H^-NMR) metabolic data acquired from peripheral blood mononuclear cells (PBMCs) taken from newly diagnosed, treatment-naïve HIV+ individuals (n = 45). PBMCs were stimulated with *M.tb*-specific antigens that elicit either a CD4^+^ (orange) or a CD4^+^ and CD8^+^ (CD4^+^/CD8^+^; purple) T cell response. Principal component 1 (PC1) explains 43.3% of the variation, while principal component 2 (PC2) explains 17.4% of the variation. (**B**) The partial least-squares discriminant analysis (PLS-DA) cross-validation graph. The PLS-DA graphic shows the distinction between CD4^+^ and CD4^+^/CD8^+^ T cells.

**Figure 2:**
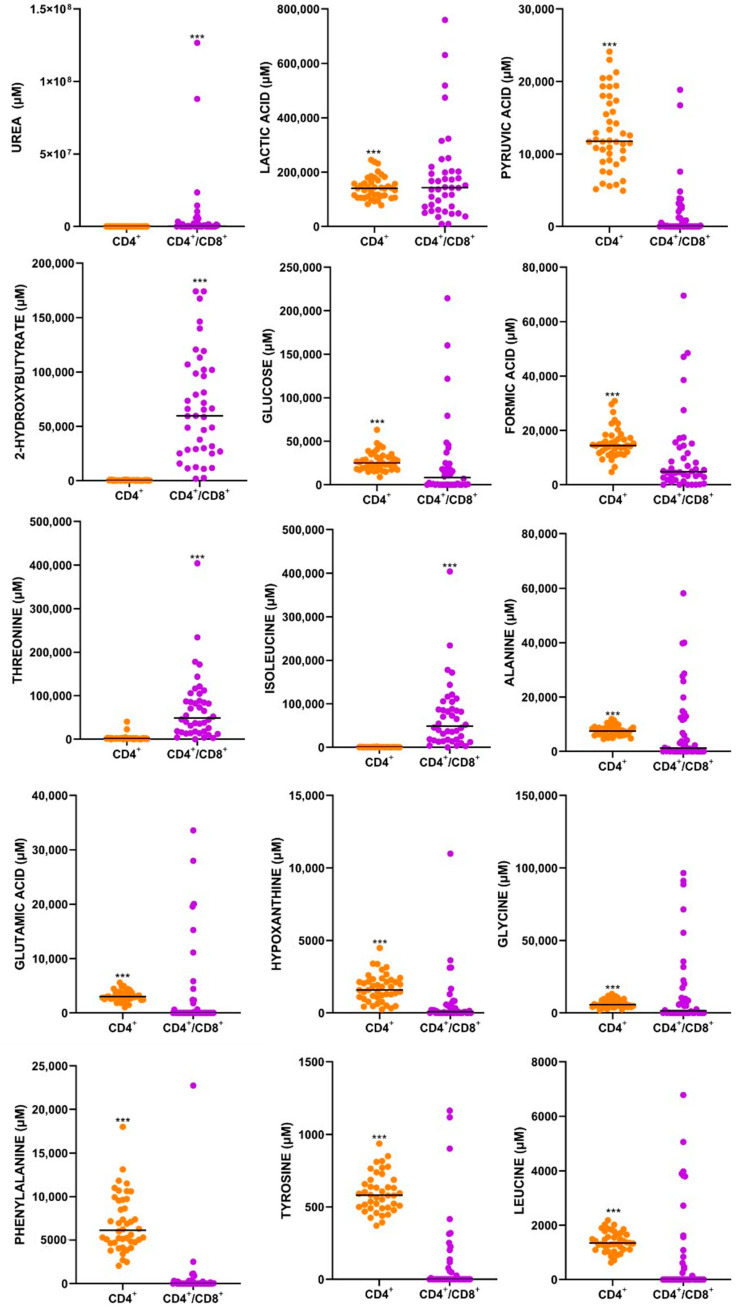
The CD4^+^ T-cell response to *M.tb* is marked by higher metabolite levels than both CD4^+^/CD8^+^ T-cell responses. The column dot plots show the metabolite levels of the fifteen most differentially expressed metabolites, quantified by 1H-NMR, between PBMCs stimulated with *M.tb*-specific antigens inducing a CD4^+^ T-cell response (orange) or a CD4^+^ and CD8^+^ (CD4^+^/CD8^+^; purple) T-cell response, respectively. FDR pairwise differences are shown (*** *p* < 0.001). The thick horizontal line on each metabolite represents the average metabolite levels.

To assess the immune function of newly diagnosed, treatment-naïve individuals infected with HIV (HIV+) in response to *M.tb*, the metabolic signature of PBMCs following 24 h of stimulation with the specific *M.tb* antigens, activating either a CD4^+^ T cell only or CD4^+^ and CD8^+^ (CD4^+^/CD8^+^) response, was captured using 1^H^ NMR spectroscopy. For dimensionality reduction and data exploration, PCA was performed (Figure 1a). PCA showed distinct clustering in the metabolic response induced by CD4^+^ T cells only, compared to CD4^+^/CD8^+^ T cells, to *M.tb*. Principal component 1 (PC1) explained 43.3% of the variance, and component 2 (PC2) explained 17.4% of the variance in the dataset (Figure 1a). To identify the metabolites contributing the most to the observed clustering, the supervised PLS-DA model was applied (Figure 1b) and cross-validated using the 10-fold CV cross-validation method. Based on the Variable Importance in Projection (VIP) plot, the top 15 metabolites contributing to the cell-type-specific immune response to *M.tb* were identified. Metabolite identification revealed urea, lactic acid and pyruvic acid (Table 1) as metabolites that significantly distinguish between the two groups. Notably, the levels of all the top 15 metabolites of importance were significantly higher (*p* < 0.001) in the majority (11) of the cases where only the CD4^+^ T-cell response was activated compared to those where both CD4^+^/CD8^+^ T cells were stimulated (Figure 2).

### 3.2. Pathways Involved in the CD4^+^ T-Cell Only, and a CD4^+^ Plus CD8^+^ T-Cell Response to M.tb

**Figure 3:**
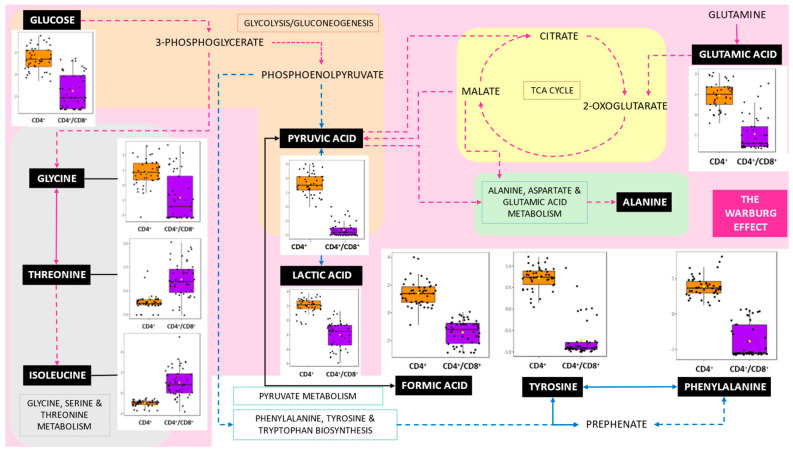
The Warberg effect and related pathways. The pink area represents the Warburg effect and includes all associated pathways, while the white area denotes pathways indirectly linked to the Warburg effect. The grey area represents glycine, serine and threonine metabolism, the orange area represents glycolysis, the yellow area represents the TCA cycle and the green area represents alanine, aspartate and glutamic acid metabolism. Black text boxes indicate metabolites identified in this study with significant variable importance in projection (VIP) chemical shifts (*p* < 0.050) between CD4^+^/CD8^+^ and CD4^+^ T cell responses. The box plots display the normalised concentration of each metabolite, with the median line representing the average concentration.

MetaboAnalyst v6.0 was employed for enrichment and pathway analysis of metabolites identified by Bayesil in T-cell responses. The analysis was conducted using relevant metabolite set libraries, including metabolic pathways, disease associations, and SNP-related metabolite sets. Both metabolite set enrichment analysis (MSEA) and quantitative enrichment analysis (QEA) were performed. MSEA provided insights into biologically meaningful patterns, facilitating functional interpretation of the metabolomic data. QEA was conducted by uploading a metabolite concentration table and analysed using the global test package. A generalised linear model was applied to compute a Q-statistic for each metabolite set, reflecting the correlation between metabolite concentrations and response outcomes. Several metabolic pathways were identified as significant, and key pathways selected for further investigation are shown in Figure 3, highlighting primary metabolites from the VIP chemical shifts in bold.

### 3.3. Impact of LTB Status on IFN-γ Levels and Metabolite Profiles in Newly Diagnosed HIV+ Individuals

The LTB status of HIV+ individuals (n = 51) was determined using the QFT-Plus interpretation workflow. Of these, 56.3% tested positive, 43.7% tested negative for LTB, and three individuals with indeterminate results were excluded, reducing the sample size to 48. In response to *M.tb* antigens ESAT-6 and CFP-10, the average IFN-γ concentration (IU/mL) was higher in the LTB+ group compared to the LTB- group for both CD4^+^ (1.29 IU/mL vs. 0.02 IU/mL) and CD4^+^/CD8^+^ T-cell responses (1.87 IU/mL vs. 0.03 IU/mL). Within the LTB+ group, IFN-γ concentrations were 45.05% higher in the CD4^+^/CD8^+^ response compared to the CD4^+^ response. In the LTB- group, the IFN-γ concentration was 24.44% higher in the CD4^+^/CD8^+^ response compared to the CD4^+^ response. An independent samples *t*-test was performed to assess whether metabolite concentrations in the CD4^+^ and CD4^+^/CD8^+^ T-cell responses were influenced by LTB status. The average chemical shift (ppm) for each metabolite varied between the two T-cell responses, with two metabolites showing significant differences.

### 3.4. Majority of the Individuals Were Vitamin D-Deficient and VDR-Methylated

**Figure 4:**
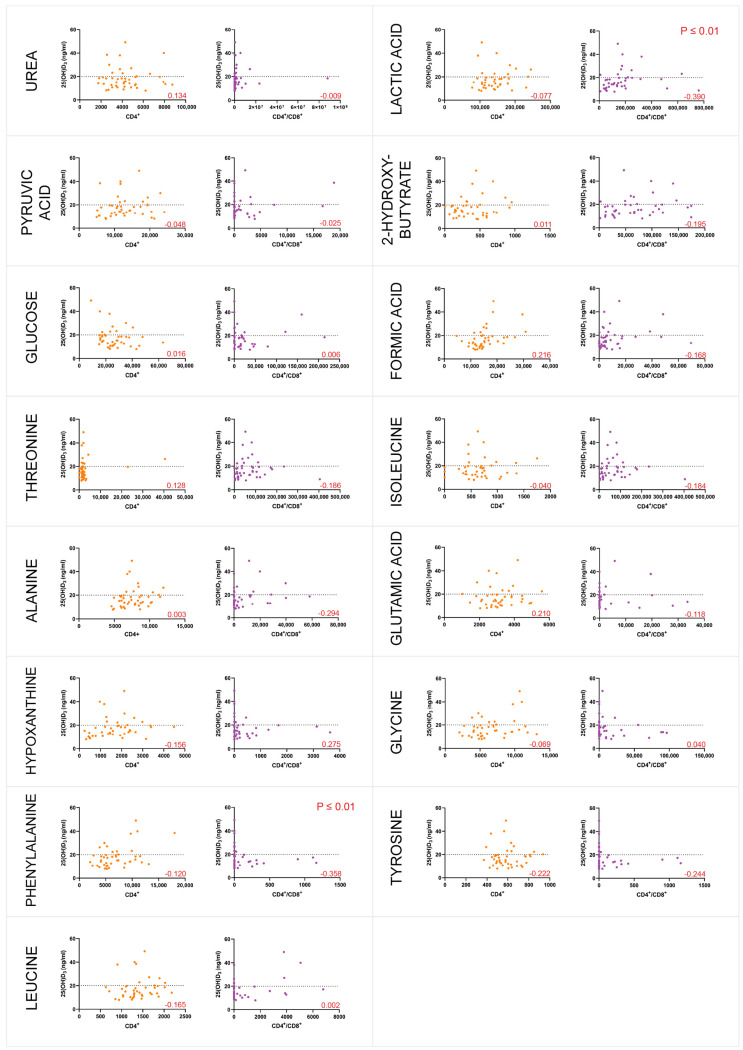
The 25-hydroxyvitamin D3 (25(OH)D_3_) concentration showed a very weak to no correlation with the metabolite levels. The scatter plots depict the association between the concentration of 25(OH)D_3_ and the amounts of metabolites in CD4^+^ (orange) and CD4^+^/CD8^+^ (purple) T cell responses. The Spearman Rho correlation coefficient is shown in the bottom right corner (red) to illustrate the effect size (R^2^ < 0.25-very weak; 0.25 < R^2^ < 0.50-weak; 0.50 < R^2^ < 0.75-moderate, R^2^ > 0.75-substantial).

**Figure 5:**
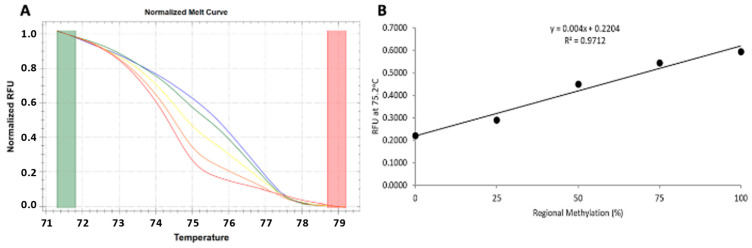
High-resolution melting normalised melt curve and standard curve for a mixture of methylated and unmethylated DNA at CGI1060 of the vitamin D receptor gene. (**A**) The normalised melt curve shows the melt profile of different mixtures of methylated and unmethylated DNA that was used to construct the standard curve (0% red, 25% orange, 50% yellow, 75% green, 100% blue). (**B**) The R2 value of 0.9712 showed a strong association between the percentage methylated DNA and the melt profile.

Given that 1,25(OH)_2_D_3_ impacts immune function [38] and that 25(OH)D_3_ deficiency has been related to LTB progression to active TB [39], participants’ plasma 25(OH)D_3_ levels were measured (n = 48) using an ELISA assay to estimate vitamin D deficiency. The standard curve had an acceptable R2 value of 0.994, allowing quantification of 25(OH)D_3_ concentration. The 25(OH)D_3_ concentration was considered deficient if the value was less than 20 ng/mL [40]. The mean 25(OH)D_3_ concentration fell within the deficient range at 18.61 ng/mL, with the majority of the participants classified as deficient (79%). Two individuals had an invalid 25(OH)D_3_ reading and were excluded from the statistical analysis. The Spearman correlation was computed to assess if plasma 25(OH)D_3_ concentration was a confounding variable in the metabolites produced by the CD4^+^ and CD4^+^/CD8^+^ T-cell responses. There was a very weak significant correlation between 25(OH)D_3_ concentration and some metabolites in the CD4^+^/CD8^+^ T-cell response, but no significant correlation was observed in the CD4^+^ T-cell response (Figure 4). To investigate the relationship between VDR methylation and HIV/*M.tb* co-infection, methylation-sensitive high-resolution melt analysis (MS-HRM) of the CGI 1060 was performed. A normalised melt curve was created by combining different ratios of human methylation and unmethylated DNA (0%, 25%, 50%, 75% and 100%), which produced a standard curve with an acceptable R2 value of 0.9712 (Figure 5). Agarose gel electrophoresis of the qPCR products demonstrated primer specificity for VDR CGI 1060.

## 4. Discussion

Despite years of research on the cellular immune response to HIV and *M.tb*, pathology and cell behaviour in the context of HIV/*M.tb* co-infection remains poorly understood. Previous research has mainly focused on HIV and active TB coinfection. This study identifies key metabolic changes in CD4+ T cells responding to *M.tb* antigens in HIV+ individuals, emphasising the role of the Warburg effect. Nine of the fifteen significantly altered VIP metabolites (glucose, glycine, glutamic acid, pyruvic acid, lactic acid, formic acid, alanine, tyrosine, and phenylalanine) are linked to this metabolic reprogramming. The Warburg effect, characterised by increased glucose uptake and lactate production via glycolysis, supports HIV replication and immune evasion.

### 4.1. Metabolites

Glucose, pyruvic acid, lactic acid, glutamic acid, alanine, glycine and phenylalanine were found to be significantly higher (*p* ≤ 0.001) in the CD4^+^ T-cell response to *M.tb* antigens in newly diagnosed HIV+ patients (Figure 2).

#### 4.1.1. Glucose

Glucose is the precursor to multiple metabolic pathways, including glycolysis, which anaerobically produces pyruvic acid and lactic acid as end products, and gluconeogenesis [41]. Glucose is also a key component in pyruvate metabolism, the process that converts glucose into pyruvic acid [42]. Additionally, it is a precursor to the Warburg effect, a process in which glucose is converted to lactic acid even in the presence of oxygen, commonly referred to as aerobic glycolysis [43]. HIV+ CD4^+^ T cells consume high levels of glucose, which can have various effects on the body [4]. Increased glycolysis in CD4^+^ T cells is associated with immune activation and low CD4^+^ T-cell counts [44]. HIV is marked by a low CD4^+^ T-cell count, suggesting that the reduced number of CD4^+^ T cells might result from their active engagement in glycolysis. CD4^+^ T cells express several glucose transporters, including Glut1, Glut3, Glut6, and Glut8 [45]. Glut1 is particularly essential for activated CD4^+^ T cells and plays a crucial role in regulating immunologic diseases [45]. Dysfunctional glucose metabolism in CD4^+^ T cells is a characteristic feature of HIV+ infection [44]. High glucose consumption by CD4^+^ T cells at sites of viral replication may significantly reduce glucose availability for CD8^+^ T cells [46]. This may explain why glucose levels were significantly higher in the CD4^+^ T-cell response to *M.tb* antigens in the presence of HIV compared to both CD4^+^ and CD8^+^ T-cell responses (Figure 2).

#### 4.1.2. Pyruvic Acid

Glycolysis, glutaminolysis [47] and pyruvate metabolism [48] are the primary metabolic pathways activated to support HIV replication [47,48]. Research indicates that CD4^+^ T cells with elevated levels of oxidative phosphorylation and glycolysis are more susceptible to HIV infection [46]. As mentioned above, pyruvic acid is the end product of glycolysis. During T-cell activation, the production of pyruvic acid is crucial for epigenomic remodelling and the effector functions of CD4^+^ T cells, although its subsequent transport into the mitochondria is not essential [49]. T cells primarily rely on oxidative phosphorylation in the mitochondria for their energy needs, utilising fatty acids, amino acids, and pyruvate [50]. Pyruvic acid causes an increase in the cytokines IL-6 and TNF-α [51]. IL-6 and TNF-α are pro-inflammatory cytokines that remain persistently elevated in the plasma of individuals with untreated HIV infection [52]. This suggests that, in this dataset, the significant increase in pyruvic acid in CD4^+^ T cells in response to *M.tb* antigens is associated with newly diagnosed (untreated) HIV infection.

#### 4.1.3. Lactic Acid

Lactic acid is produced though the metabolic process of glycolysis [53,54] from pyruvic acid [53], also identified in this dataset. Lactic acid can affect CD4^+^ T cells in various ways, including by increasing IL-17 production [55], inhibiting motility [55] and modulating polarisation [56]. Lactic acid also acts as an alternate carbon source for T cells supporting its metabolism and activation [57,58].

Lactic acid accumulation triggers the uptake of the lactate transporter SLC5A12 by CD4^+^ T cells, resulting in an increase in the proinflammatory cytokine IL-17 production and enhanced fatty acid synthesis [55]. By enhancing fatty acid synthesis, lactic acid induces a metabolic reprogramming of cells [56] and inhibiting of T-cell mobility [50]. Lactic acid also inhibits CD4^+^ T-cell motility by interfering with glycolysis, which is essential for CD4^+^ T cells to migrate to sites of infection [55,59]. This interference not only results in the production of high amounts of IL-17 but also triggers the loss of cytolytic activity [54]. Lactic acid induces polarisation of the effector phenotype of CD4^+^ T cells, which can also lead to more IL-17 and IL-2 production [59,60] and results in increased IFN- γ production in CD8^+^ T cells [55], which is the key mediator required to activate macrophages to limit *M.tb* growth.

In an HIV+ environment, IL-17A assists HIV infection by reprogramming intestinal epithelial cells to facilitate HIV transmission into CD4^+^ cells [61]. CD4^+^ T cells are the well-established targets of HIV infection. IL-17 works together with IFN- γ to contribute to granuloma formation in response to *M.tb*. IL-2 is a T-cell growth factor that plays a role in the proliferation of CD4^+^ T cells [62]. Lactic acid has the potential to negatively impact CD8^+^ T-cell activation, leading to significantly reduced proliferation and lower cell numbers [57]. This may explain why lactic acid was observed to be significantly higher in this dataset in the CD4^+^ T-cell response (Figure 2).

In addition, a statistically significant correlation coefficient of −0.390 with a *p*-value less than 0.01 was found in the CD4^+^/CD8^+^ T-cell response to lactic acid and 25(OH)D_3_ concentration levels (Figure 4). This correlation coefficient indicates a reliable moderate negative relationship between 25(OH)D_3_ concentration and lactic acid. Lactate dehydrogenase (LDH), also known as lactic acid dehydrogenase, is an enzyme that catalyses the reversible conversion of pyruvate to lactate and lactate to pyruvate [63]. Higher concentrations of 25(OH)D_3_ may regulate the production of key muscle proteins, such as lactic acid dehydrogenase [64]. As a result, a deficiency in 25(OH)D_3_ may lead to an accumulation of lactic acid in the muscles, which could explain the findings observed in this study.

#### 4.1.4. Glutamic Acid

Glutamine is the primary source of intracellular glutamic acid and serves as the primary substrate in the citric acid cycle [65]. Glutamic acid is secreted by HIV-infected CD4^+^ T-cells, and glutamine metabolism plays a crucial role in regulating CD4^+^ T-cell proliferation and HIV infection [65]. HIV infection of macrophages and overexpression of HIV viral protein R (Vpr) induce significant changes in multiple metabolic pathways, leading to increased glutamate production and release through the activation of glycolysis and the TCA cycle [66]. HIV Vpr mediates various processes that facilitate HIV infection and promote its persistence in host cells [67,68]. Therefore, the increase in glutamic acid production in HIV infection suggests that it facilitates HIV infection and persistence in host cells.

#### 4.1.5. L-Alanine

L-alanine is an amino acid essential for T-cell activation and the restimulation of memory T cells [69]. T cells utilise L-alanine to support protein synthesis, which occurs in large quantities upon T-cell activation [69]. Viral protein U (Vpu) is a small protein encoded by human immunodeficiency virus type 1 (HIV-1) that plays a key role in regulating HIV infection and transmission [70]. HIV-1 Vpu reduces the cellular uptake of L-alanine by downregulating the L-alanine transporter SNAT1, which in turn prevents CD4^+^ T-cell mitogenesis [71]. Since CD4^+^ T cells stimulate CD8^+^ T cells [72], this suggests that HIV weakens the immune system by inhibiting the use of L-alanine to promote CD4^+^ T-cell production, which in turn affects CD8^+^ T-cell activation.

#### 4.1.6. Glycine

Glycine plays a crucial role in the development and activation of T cells, as well as in the regulation of CD4^+^ T-cell responsiveness [73]. Glycine has not been studied in in-depth relation to HIV; however, glycine is involved in the metabolism of *M.tb*. Glycine is one of the amino acids used by *M.tb*, playing a role in its nitrogen metabolism and contributing to the pathogen’s survival and proliferation within human host cells [74]. This may explain why a significant difference was seen in the glycine levels between LTB+ and LTB- individuals in the CD4^+^/CD8^+^ T-cell response.

#### 4.1.7. L-Phenylalanine

In this dataset, there was a negative correlation between L-phenylalanine and 25(OH)D_3_ concentration in the CD4^+^/CD8^+^ T-cell response (Figure 4). Elevated levels of l-phenylalanine are associated with HIV disease progression, specifically correlating with lower CD4^+^ T-cell counts [75]. These findings suggest that the interaction between vitamin D levels and amino acid metabolism could play a critical role in immune function, potentially affecting the severity and progression of HIV. Further studies exploring the mechanisms underlying this relationship may provide valuable insights into the metabolic changes observed in HIV+ patients and highlight potential therapeutic targets for improving immune responses in this population.

### 4.2. Pathways

#### 4.2.1. The Warburg Effect, Gluconeogenesis, Glutamate and Pyruvate Metabolism

Glucose, pyruvic acid, lactic acid and glutamic acid are linked to the Warburg effect; glucose, pyruvic acid, and lactic acid are linked to gluconeogenesis; pyruvic acid, glutamic acid and glycine are linked to glutamate metabolism, and pyruvic acid and lactic acid are linked to pyruvate metabolism. To the best of our knowledge, the Warburg effect has primarily been studied in the context of cancer research [43,76,77]. The Warburg effect is thought to function as a metabolic switch, influencing the host’s immune response [78]. HIV uses metabolic programming to gather free nucleotides, lipids, and amino acids for viral replication and assembly [46]. The Warburg effect is essentially a metabolic process in which cells take up more glucose and produce more lactic acid, thereby increasing their reliance on glycolysis instead of oxidative phosphorylation to generate energy [79]. This study indicates that these metabolites reflect significant metabolic reprogramming in the CD4^+^ T-cell response to *M.tb* antigens in HIV+ individuals. This reprogramming is consistent with the Warburg effect, contributing to immune dysregulation and highlighting potential metabolic targets for therapeutic intervention. Gluconeogenesis is a process that takes place in the liver and kidneys to generate glucose during periods between meals [80]. Therefore, gluconeogenesis generates glucose that is utilised in HIV/*M.tb* co-infection through the Warburg effect. HIV Vpr not only facilitates HIV infection but also contributes to the long-term persistence of the virus in macrophages [66]. The activation of glycolysis results in increased production and release of glutamic acid, a process that is further amplified by HIV Vpr [66]. This metabolic shift indicates that glutamate metabolism, driven by the Warburg effect, plays a role in immune dysregulation and supports the persistence of HIV in host cells in response to *M.tb* antigens ESAT-6 and CFP-10.

#### 4.2.2. Amino Sugar Metabolism

Glutamic acid and pyruvic acid are also linked to the amino sugar metabolic pathway. Acetic acid and glutamine were identified in the overall dataset as significant between CD4^+^ and CD4^+^/CD8^+^ T cells and are also part of this pathway. Amino sugar metabolism involves the process of metabolising chemical compounds that have a sugar backbone, with an amino group replacing one of the hydroxyl groups [81]. The envelope glycoproteins of HIV mediate the cellular uptake of the virus and the fusion of infected and non-infected CD4^+^ T cells [82]. Amino sugar derivatives may possess potential anti-HIV activity by inhibiting glycoprotein processing [83]. This suggests that amino sugar metabolism was found to be significant in this dataset due to the increase in HIV in the newly diagnosed sample set and its potential to inhibit HIV envelope glycoproteins on the virus’s surface.

### 4.3. Potential Therapeutic Targets

Given the metabolic alterations observed in this study, specific metabolic pathways are proposed as potential therapeutic targets. Since increased glycolysis in CD4^+^ T cells is linked to immune activation and HIV replication, targeting this pathway could provide therapeutic benefits [73]. The use of 2-deoxyglucose (2-DG), an inhibitor of glycolysis, or pyruvate kinase M2 (PKM2) modulators [84], could help regulate excessive immune activation seen in HIV and *M.tb* infections. Elevated glutamic acid levels suggest disruptions in glutamine metabolism [85]. Glutaminase inhibitors (e.g., CB-839) could be used to modulate T-cell metabolism, potentially reducing hyperactivation while preserving immune function. The increase in lactic acid highlights the role of glycolysis in immune evasion. Targeting lactate transporters (e.g., SLC5A12 inhibitors) could help prevent excessive immune suppression and reprogram T-cell function. While many of these metabolic inhibitors, such as 2-DG and CB-839, have been explored in cancer and immune disorders, their application in infectious diseases like HIV/*M.tb* co-infection remains experimental. All of these pathways are linked to the Warberg effect. Additional studies incorporating targeted metabolomics, proteomics, and in vivo models are required to confirm the efficacy of these interventions and determine their impact on immune function.

### 4.4. Vitamin D Deficiency and VDR Methylation

Most of the participants in this study had 25(OH)D_3_ deficiency, which was consistent with previous studies on HIV and vitamin D deficiency [86,87]. Vitamin D is essential for cell growth, immunity, and metabolism, and vitamin D deficiency is widespread in both developed and developing countries [88]. HIV affects the immune system, and HIV+ individuals generally have a significantly lower 25(OH)D_3_ concentration [89]. Additionally, in immune-mediated diseases, 25(OH)D_3_ and 1,25_2_(OH)D_3_ are critical for inhibiting T-cell proliferation [90]. HIV targets CD4^+^ T cells for proliferation, and a deficiency in 25(OH)D_3_ suggests that HIV-infected CD4^+^ T-cell proliferation may not be successfully inhibited. Most of the samples in this study were methylated in the VDR CGI 1060 region, which could be attributed to the participant’s HIV+ status. HIV-induced methylation reduces VDR expression in the promoter region at the 5′ end [91]. Glutamic acid, l-tyrosine, urea and hypoxanthine all had significant differences in the CD4^+^ T-cell response between methylated and unmethylated groups. All four metabolites had a negative correlation. This suggests that, as the VDR gene methylation increases, these metabolite levels tend to decrease, or conversely higher metabolite levels are associated with lower methylation rate. Since glutamic acid is involved in the Warberg effect, as mentioned above, VDR methylation may influence the expression of genes involved in glutamine metabolism or related pathways. Further investigation is needed to determine the precise mechanisms by which VDR methylation modulates glutamine metabolism and its impact on HIV/*M.tb* co-infection.

### 4.5. Limitations

In addition to some individuals declining to participate in the study, the clinic was less busy than expected on most sampling days, which led to a small sample size. While not a large number, it allowed for the identification of distinct metabolic profiles associated with different T-cell responses. This provides a foundation for future, larger-scale studies. Prior to this work, there was a lack of systematic investigation into the metabolic interplay between these cell types in this context. The study’s findings, even if preliminary, offer a valuable starting point for future research. Additionally, the lack of HIV-negative samples limits the study’s ability to provide a more comprehensive comparison for analysis.

## 5. Conclusions

This study aimed to assess the role of CD8^+^ T cells in the immune response to *M.tb* antigens in a newly diagnosed, HIV+ environment. Contrary to expectations, the findings suggest that CD4^+^ T cells play a more dominant role in the immune response, likely due to the untreated HIV status of the participants. This was consistent with existing literature that found CD4^+^ T cells to be more active in an untreated HIV+ environment [92]. The results indicate that HIV may exploit metabolic pathways, such as the Warburg effect, to facilitate its entry into CD4^+^ T cells, further influencing immune function

Notably, the Warburg effect was observed for the first time in relation to HIV infection in response to *M.tb* antigens ESAT-6 and CFP-10. IFN-γ levels remained comparable between CD4^+^ and CD8^+^ responses, suggesting that while both T-cell subsets contribute to the immune response, their metabolic and functional roles may differ in the context of early HIV infection.

## Figures and Tables

**Table 1:** Metabolites from the variable importance in projection (VIP) plot in the partial least-squares discriminant analysis (PLS-DA) model distinguishing between the immune response of CD4^+^ T cells and CD4^+^/CD8^+^ T cells to *M.tb* antigens.

Metabolite	Synonym ^1^	Confidence Score ^2^	*p*-Value ^3^	FDR ^3^
Urea		8	2.6665 × 10^−12^	6.9329 × 10^−12^
L-Lactic Acid	Lactic Acid	10	9.5153 × 10^−22^	5.3014 × 10^−21^
Pyruvic Acid		8	2.8772 × 10^−40^	1.1221 × 10^−38^
2-Hydroxybutyrate		9	3.2643 × 10^−24^	2.1218 × 10^−23^
D-Glucose	Glucose	10	1.2451 × 10^−16^	4.8559 × 10^−16^
Formic Acid		9	1.049 × 10^−25^	1.0228 × 10^−24^
L-Threonine	Threonine	9	5.1955 × 10^−12^	1.2664 × 10^−11^
L-Isoleucine	Isoleucine	7	1.717 × 10^−14^	6.0875 × 10^−14^
L-alanine	Alanine	10	1.0802 × 10^−12^	3.5108 × 10^−12^
L-Glutamic Acid	Glutamic Acid	10	7.1441 × 10^−20^	3.4828 × 10^−19^
Hypoxanthine		10	8.7547 × 10^−25^	6.8287 × 10^−24^
Glycine		10	1.1789 × 10^−8^	2.5542 × 10^−8^
L-Phenylalanine	Phenylalanine	10	9.1936 × 10^−32^	1.1952 × 10^−30^
L-Tyrosine	Tyrosine	10	7.9541 × 10^−33^	1.551 × 10^−31^
L-Leucine	Leucine	10	4.2008 × 10^−17^	1.8203 × 10^−16^

^1^ Obtained from HMDB. ^2^ The confidence scores were obtained from Bayesil. ^3^ The *p*-value and false discovery rates (FDR) were using the *t*-test in MetaboAnalyst.

## Data Availability

The original contributions presented in this study are included in the article/Supplementary Material. Further inquiries can be directed to the corresponding author(s).

## Supplementary Materials

The following supporting information can be downloaded at: https://www.mdpi.com/article/10.3390/metabo15050285/s1, Section S1; Table S1. Comparing Significant metabolite levels in CD4+ and CD4+/CD8+ T-cell responses between the old and new data seta.

## Abbreviations

The following abbreviations are used in this manuscript:

2-DG	2-deoxyglucose
APC	Antigen Presenting Cells
BCG	Bacillus Calmette–Guérin
CD4+	Cluster of Differentiation 4-Positive
CD8+	Cluster of Differentiation 8-Positive
CFP-10	Culture Filtrate Protein 10
dH_2_O	Distilled Water
ESAT-6	Early Secreted Antigenic Target 6
HIV	Human Immunodeficiency Virus
IGRA	Interferon Gamma Release Assay
INF-γ	Interferon Gamma
LTB	Latent Tuberculosis
*M.tb*	*Mycobacterium tuberculosis*
MBC	Minimum Bactericidal Concentration
MHC	Major Histocompatibility Complex
MIC	Minimum Inhibitory Concentration
MS-HRM	Methylation-Sensitive High-Resolution Melt Analysis
NMR	Nuclear Magnetic Resonance
PBMCs	Peripheral Blood Mononuclear Cells
PBS	Phosphate Buffer Solution
PKM2	Pyruvate Kinase M2
QFT	QuantiFERON^®^
TSP	Trimethylsilyl-2,2,3,3-Tetradeuteropropionic Acid
TXI	Triple Resonance Inverse
VDR	Vitamin D receptor
