# Supplementary material for: Metabolic Reprogramming in HIV+ CD4+ T-Cells: Implications for Immune Dysfunction and Therapeutic Targets in M. tuberculosis Co-Infection
Source: Metabolites. 2025 Apr 22;15(5):285. doi: 10.3390/metabo15050285 (PMC12112849; doi:10.3390/metabo15050285)
Supplement: Supplementary file 1 [file metabolites-15-00285-s001.zip › metabolites-3547410-supplementary.pdf]

Section S1

1.1. Validation of $CD4^+$ and $CD4^+/CD8^+$ T-cell response result

To ensure that the observed differences in T-cell responses were not due to instrumentation variability, 18 separate samples were analysed using the same instrument and protocol. Multiple metabolites were confirmed to have the same difference between the $CD4^+$ T-cell response and the $CD4^+/CD8^+$ T-cell response. These results are compared to the previous results in table S1.

1.2. ***Sample Preparation and spectral analysis***

To prepare the buffer, 7.85g of anhydrous Na_2HPO_4 and 0.230g of DSS were measured and mixed in 100ml of distilled water. The pH was measured and adjusted to between 6.8 and 7.5 using NaOH or HCl. For sample preparation, the frozen plasma samples were thawed at room temperature, and Amicon filters were rinsed seven times with distilled water. A volume of 285µl of the plasma sample was placed in the filter and centrifuged at 12,000g at 4°C. The sample was then transferred to NMR tubes, with 35µl of D_2O and 30µl of buffer added to achieve a final volume of 350µl. Additionally, 100 µM (0.0001g) of formate was added to the NMR tubes. The pH was adjusted to approximately 7.0 with NaOH or HCl as needed. Including 100 µM formate provided a downfield signal that enhanced Bayesil's phasing results during automatic spectral processing, as poorly phased spectra could lead to quantification errors. Since formate can be present in some biological samples, 100 µM was subtracted from the reported value.

For spectral analysis, “2D NOESY” was typed in the spectral analysis box, ensuring the temperature was set to 25°C with a 12 ppm sweep width, 4 s acquisition time, 100 ms mixing time, 10 ms recycle delay, and 990 ms saturation delay. Eight steady scans were run, calibrating the pre-saturation pulse to between 60-80 Hz. The water peak was identified by typing “pulsenoesypr1d”; the peak was observed around 4.78 ppm, and the spectra were calibrated to the water peak. The excitation pulse was calibrated to a 90° flip angle, and the analysis was run. Finally, the data were loaded into Bayesil software, and the peaks were checked after selecting the boxes for plasma filtered, DSS, and 500 MHz.

Table S1. Comparing Significant metabolite levels in CD4$^+$ and CD4$^+$/CD8$^+$ T-cell responses between the old and new data set[a]

**METABOLITE**	CONFIDENCE SCORE	P-VALUE (OLD DATA SET)	P-VALUE (NEW DATA SET)
**UREA**	**8**	**2.6665E-12**	**0,046**
**L-LACTIC ACID**	**10**	**9.5153E-22**	**0,002**
**PYRUVIC ACID**	**8**	**2.8772E-40**	**0,046**
**2-HYDROXYBUTYRATE**	**9**	**3.2643E-24**	**0,001**
**D-GLUCOSE**	**10**	**1.2451E-16**	**0,046**
**FORMIC ACID**	**9**	**1.049E-25**	**0,006**
**THREONINE**	**9**	**5.1955E-12**	**0,006**
**ISOLEUCINE**	**7**	**1.717E-14**	**0,017**
L-ALANINE	10	1.0802E-12	0,734
**L-GLUTAMIC ACID**	**10**	**7.1441E-20**	**0,006**
HYPOXANTHINE	**10**	**8.7547E-25**	**0,046**
**GLYCINE**	**10**	**1.1789E-8**	**0,002**
L-PHENYLALANINE	10	9.1936E-32	0,240
L-TYROSINE	10	7.9541E-33	0,954
L-LEUCINE	10	4.2008E-17	0,734

[a]The bold metabolites in the table remained significant in both data sets
